# Modulating tiller formation in cereal crops by the signalling function of fertilizer nitrogen forms

**DOI:** 10.1038/s41598-020-77467-3

**Published:** 2020-11-25

**Authors:** Bernhard Bauer, Nicolaus von Wirén

**Affiliations:** 1 Molecular Plant Nutrition, Leibniz-Institute of Plant Genetics and Crop Plant Research (IPK), Corrensstr. 3, 06466 Gatersleben, Germany; 2 Crop Production and Crop Protection, Institute of Biomass Research, University of Applied Sciences Weihenstephan-Triesdorf, Markgrafenstrasse 16, 91746 Weidenbach, Germany

**Keywords:** Plant sciences, Plant hormones, Cytokinin

## Abstract

Cereal crop yield comprises interrelated components, among which the number of tillers is highly responsive to nitrogen fertilization. We addressed the hypothesis of whether the supply of different nitrogen forms can be employed to manipulate the tiller number in cereal crops. Relative to urea or ammonium, exclusive supply of nitrate increased tiller number in hydroponically-grown barley plants. Thereby, tiller number correlated positively with the root-to-shoot translocation rate of endogenous cytokinins. External supply of a synthetic cytokinin analog further stimulated tillering in nitrate-containing but not in urea-containing nutrient solution. When the cytokinin analog 6-benzylaminopurine riboside was externally supplied to roots, its translocation to shoots was 2.5 times higher in presence of nitrate than in presence of urea or ammonium, suggesting that cytokinin loading into the xylem is affected by different nitrogen forms. We then translated this finding to field scale, cultivated winter wheat in four environments, and confirmed that nitrate fertilization significantly increased tiller number in a dose-dependent manner. As assessed in 22 winter wheat cultivars, nitrogen form-dependent tiller formation was subject to substantial genotypic variation. We conclude that cytokinin-mediated signaling effects of fertilizer nitrogen forms can be employed as a management tool to regulate the tiller number in cereal crops.

## Introduction

For agricultural plant production, mineral nitrogen (N) fertilizers are available in different forms, of which urea is worldwide the most widespread, followed by nitrate and ammonium^1^. From an agronomic perspective, the choice of a particular fertilizer N form is mainly determined by commercial availability, price, and potentially beneficial effects of the accompanying ions. With increasing consideration of their environmental impact, N fertilizer forms are also evaluated for their mobility in the soil, their effects on soil pH, nutrient mobilization, potential leaching or denitrification, and particularly for ammonia volatilization^2–4^. Regarding the physiology of crops, using different N forms has profound implications, since their external availability is associated with distinct root uptake and translocation rates, different intensities and sites of N assimilation, and changes in primary and secondary metabolism, which may ultimately alter plant growth and development^5,6^. In particular, studies comparing ammonium and nitrate as major or sole N sources for plants have shown that ammonium supply can decrease photosynthesis, respiration, and water use^7,8^ while increasing tolerance to drought stress and pathogens^9–11^. In the long run, developmental changes caused by exclusive ammonium supply can result in accelerated flowering, while nitrate provision stimulates vegetative growth and delays plant senescence^6^. Such obvious effects caused by different N forms are mostly observed under controlled conditions, e.g., in hydroponics, whereas N-form effects are weaker and less significant in the field, where applied N forms are not the only N source and undergo immobilization and microbial inter-conversion. Even when urea fertilizers are stabilized by urease inhibitors or ammonium fertilizers by nitrification inhibitors, microbial conversion is suppressed only in part and transiently^4,12^. Despite the efficacy of urease and nitrification inhibitors and their importance to mitigate ammonia emissions and meet N fertilizer regulations in Europe^13^, the poor predictability of the fate of defined fertilizer N forms in soils and their subsequent impact on relevant agronomic crop traits may be the major limitation for the targeted use of fertilizer N forms to control crop development.

Among the major yield components, in particular, the tiller number strongly responds to N fertilization. As a significant determinant of the grain-to-straw ratio and the grain yield, tiller number influences the use efficiency of mineral fertilizers and water^14,15^. However, tiller number mostly decreases during the generative growth phase as a result of tiller abortion, a process strongly influenced by cereal crop species/cultivar, sowing density, light quality, and climate conditions^16–18^. Tiller formation increases with N fertilizer dose and responds to the fertilizer N form, as combined ammonium nitrate enhanced tiller number more effectively than the same amount of either N form alone^19–21^. The physiological basis of this N form effect relies, at least in part, on phytohormone signaling. On the one hand, N supply prevents the biosynthesis of strigolactones, a class of root-derived phytohormones that are synthesized under N deficiency and suppress shoot branching and tiller formation when translocated to the shoots^6,22^. On the other hand, N supply, especially nitrate strongly promotes the biosynthesis and root-to-shoot translocation of cytokinins, which are predominantly synthesized in growing root meristems and required in shoots for division and expansion of leaf cells, shoot branching, and tiller formation^23–25^. While an adverse effect of ammonium nutrition on cytokinin translocation in the xylem has been shown^26,27^, the impact of ammonium or urea fertilization on cytokinin translocation and tiller formation in cereals has remained contradictory^19,28^.

In the present study, we tested the hypothesis that tiller formation in cereal crops can be manipulated by the supply of different N forms, irrespective of whether plants grow in hydroponics or the field. We first examined the response of hydroponically-grown barley plants to a pure or mixed supply of nitrate and urea that was stabilized with the urease inhibitor NBPT^4^. To verify the impact of fertilized N forms on cytokinins, xylem sap was analyzed with radio-immune assays, and translocation studies were performed with traceable artificial cytokinins. We then translated the results from our physiological experiments in artificial growth media to the field scale. In field trials with winter wheat, which has a lower tillering potential than barley^29^, we investigated under which growth conditions the different nitrogen fertilizer forms can be employed to modulate tiller number in agricultural practice.

## Results

**Figure 1:**
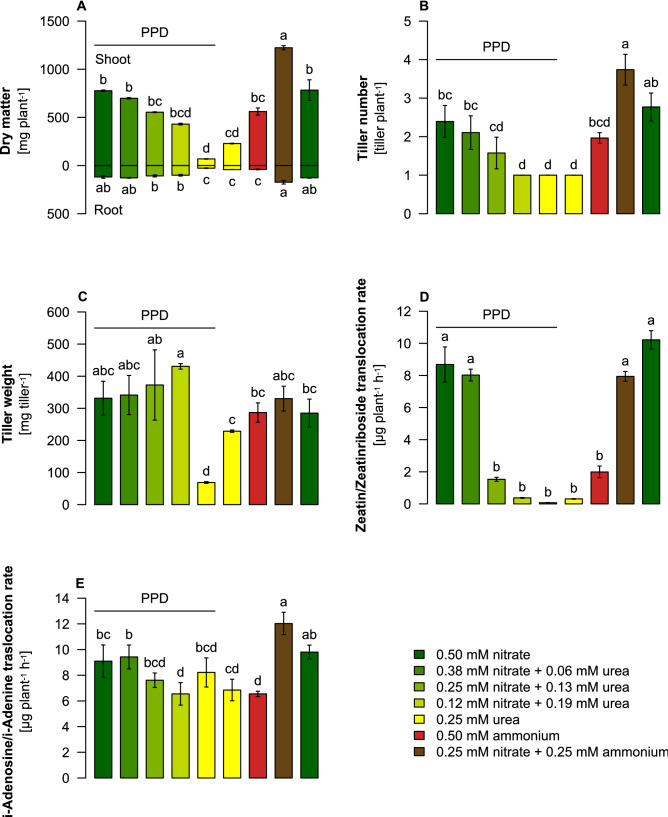
Dry weight, tiller number and cytokinin translocation rates in barley plants as affected by the supply of different nitrogen forms. (**A**) Dry weight per plant, (**B**) tiller number per plant incl. the primary shoot axis, (**C**) dry weight per single tiller, (**D**) root-to-shoot translocation rate of the cytokinin forms zeatin (Z) and zeatin-riboside (ZR) and (**E**) root-to-shoot translocation rate of the cytokinin precursors isopentenyl-adenine (i-Ade) and isopentenyl-adenosine (i-Ado) as determined in the xylem bleeding sap. Five days-old barley seedlings were transferred for 40 days to nutrient solution containing the following N sources: 0.5 mM KNO_3_, 0.38 mM KNO_3_ + 0.06 mM urea (75:25% N), 0.25 mM KNO_3_ + 0.13 mM urea (50:50% N), 0.12 mM KNO_3_ + 0.19 mM urea (25:75% N), 0.25 mM urea, 0.25 mM NH_4_NO_3_. The pH was buffered at pH 6.6 by Ca(HCO_3_)_2_, and wherever indicated 75 µg L^−1^ of the urease inhibitor PPD was added. Bars represent means ± SD, n = 3 biological replicates. Different letters indicate significant differences among treatments at p < 0.05 by Tukey’s test.

We first focused on the influence of nitrate and urea on growth and tiller formation of barley plants grown in pH-buffered nutrient solution supplemented with 0.5 mM N at different ratios of nitrate to urea. Under the exclusive supply of nitrate, barley plants grew densely, formed the highest shoot biomass, and on average, 2.7 tillers per plant (Fig. 1A,B; Supplementary Fig. 1). Stepwise replacement of nitrate-N by urea-N decreased dry weight and tiller number. Purely urea-fed plants had only a single short main shoot and no tillers. To prevent urea degradation, the urease inhibitor PPD was added, and comparing nitrate-supplied plants with or without PPD showed that its presence did not significantly influence plant growth or tiller number. Since urea and ammonium toxicity have been suggested as major causes of growth depression under the supply of reduced nitrogen forms^30^, we also measured ammonium and urea levels. Despite their increase with urea supply, neither ammonium nor urea concentrations in roots or shoots reflected closely an N treatment effect on dry weight and tiller number at elevated urea or exclusive ammonium supply (Fig. 1A,B; Supplementary Fig. 2A,B). In fact, the absence of toxicity symptoms on the leaves in the phenotypic assessment of urea- and ammonium-fed plants (Supplementary Fig. 1), as well as the dry weights of single tillers, revealed that under 75% urea-N (0.19 mM urea), the growth of individual tillers was even stronger than in purely nitrate-fed plants (Fig. 1C). Therefore, urea or ammonium toxicity in root or shoot tissues was unlikely to account solely for urea-induced depression of growth and tillering.

In tobacco and tomato, nitrate had previously been shown to stimulate the root-to-shoot translocation of cytokinins^26,27^. Here, we immune-detected the physiologically active cytokinin form zeatin and its transport form zeatin-riboside in the xylem bleeding sap of barley plants and observed that their translocation rate decreased strongly with increasing supply of urea-N (Fig. 1D). Since urea supply also repressed the amount of xylem bleeding sap collected at the hypocotyl (Supplementary Fig. 2C), we immune-detected separately the physiologically less active cytokinin forms isopentenyl-adenine and isopentenyl-adenosine. However, their translocation rates were much less affected by the form of N supply (Fig. 1E), indicating that the smaller xylem sap volume was not the primary cause for decreasing translocation rates of zeatin and zeatin-riboside.

**Figure 2:**
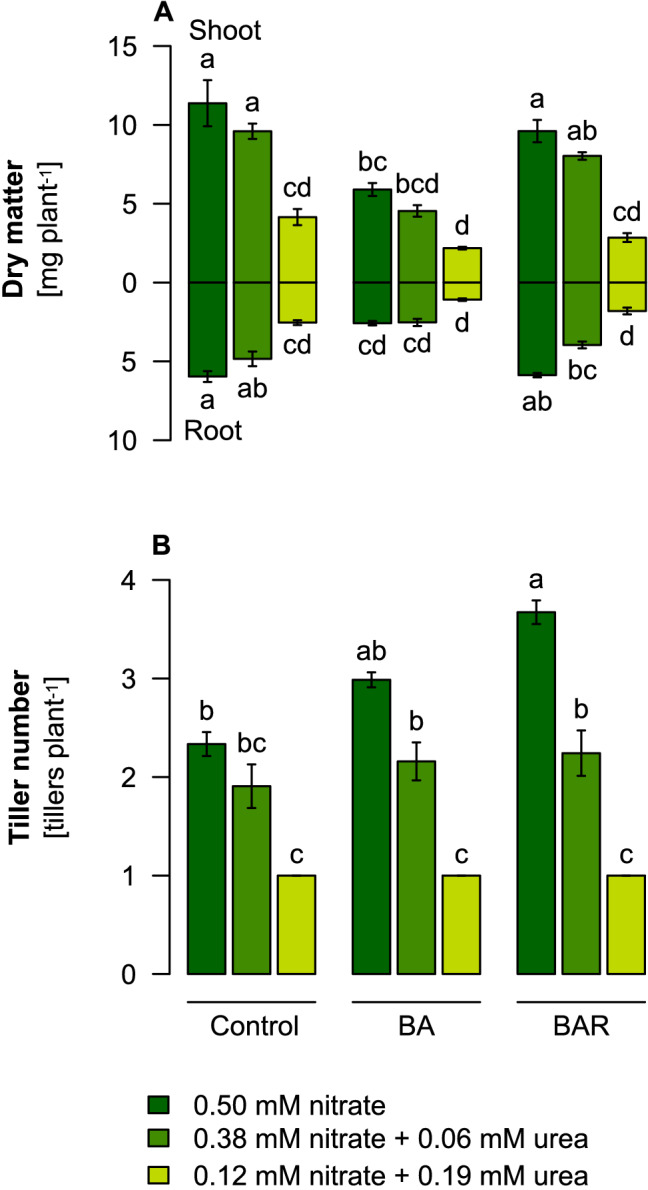
Growth and tiller formation in barley after external supplementation of cytokinins in the presence of nitrate or urea. (**A**) Dry weight of shoots and roots and (**B**) tiller number of spring barley plants precultured hydroponically on ammonium nitrate before transfer to different N sources: 0.5 mM KNO_3_, 0.38 mM KNO_3_ + 0.06 mM urea (75:25% N), or 0.12 mM KNO_3_ + 0.19 mM urea (25:75% N). The pH was buffered at pH 6.6 by Ca(HCO_3_)_2_. The cytokinin analogue 6-benzylaminopurine (BA) was supplied at a concentration of 10 µM at the beginning of tillering (3-leaf stage). Tillers were counted at the 7-leaf stage. The urease inhibitor PPD was supplemented at 75 µg L^−1^. Bars represent means ± SD, n = 20 biological replicates. Different letters indicate significant differences among treatments at p < 0.05 by Tukey’s test.

We then addressed whether suppressed tiller formation in the presence of urea can be overcome by the exogenous supply of cytokinin. When barley plants had reached the 3-leaf stage, we added the synthetic cytokinin analog 6-benzylaminopurine (BA) or its conjugated form 6-benzylaminopurine riboside (BAR) to the nutrient solution. After tiller formation had progressed and plants reached the 7-leaf stage, BA supply had reduced total shoot and root dry weights to a similar extent in all N treatments (Fig. 2A). Notably, only in plants exclusively supplied with nitrate, BA or BAR supply enhanced tiller number slightly or significantly, respectively, relative to control plants (Fig. 2B). However, in the presence of 25% or 75% urea-N no additional tillers were formed. This observation suggested that urea suppresses tiller formation by inhibiting either BA uptake from the external medium or its root-to-shoot translocation.

**Figure 3:**
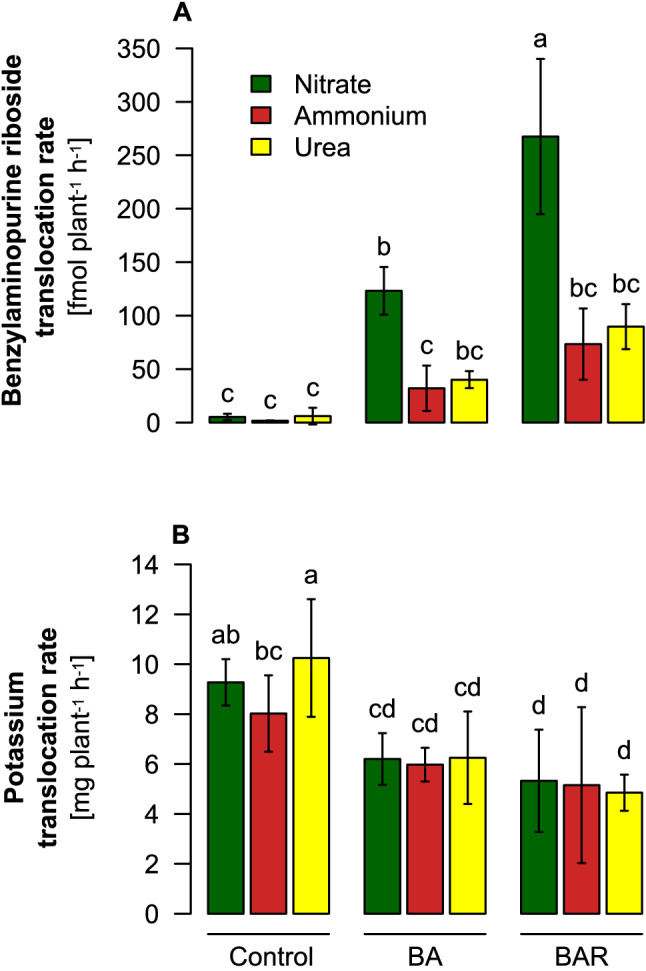
Translocation rate of benzylaminopurine riboside (BAR) and potassium in the xylem sap of barley plants as affected by the presence of different nitrogen forms. (**A**) Benzyl-aminopurine translocation rate and (**B**) potassium translocation rate as determined in the xylem bleeding sap from spring barley precultured hydroponically until the 5-leaf stage on ammonium nitrate, then for 24 h on N-free nutrient solution and then for 48 h on pH-buffered 0.5 mM potassium nitrate, urea or ammonium sulfate. BAR was supplied at a concentration of 10 µM 12 h before collection of the xylem bleeding sap. Bars represent means ± SD, n = 3 biological replicates. Different letters indicate significant differences among treatments at p < 0.05 by Tukey’s test.

Previously, the conjugated cytokinin analog BAR has been shown to be readily taken up by roots and promote tiller bud outgrowth in rice^5^. We thus verified the N form´s impact on BAR translocation in a short-term study, in which BAR was supplemented to plants that had been acclimated to either nitrate, ammonium, or urea for 36 h. When xylem bleeding sap was collected 12 h after BAR supply to roots and assayed specifically for BAR by ELISA, the BAR translocation rate was 2.5 times higher in the presence of nitrate than in the presence of the reduced N forms ammonium or urea (Fig. 3A). Negligible BAR levels in plants not supplied with BAR confirmed specificity of the antibody used in the ELISA and excluded cross-reactivity with endogenous cytokinins. To account for treatment effects on xylem exudation, K translocation rates were determined and found to be lower, especially after the external supply of BAR, but not differentially affected by either N form (Fig. 3B). Thus, this experiment indicated a stimulatory effect of nitrate on the root-to-shoot translocation of cytokinins.

**Figure 4:**
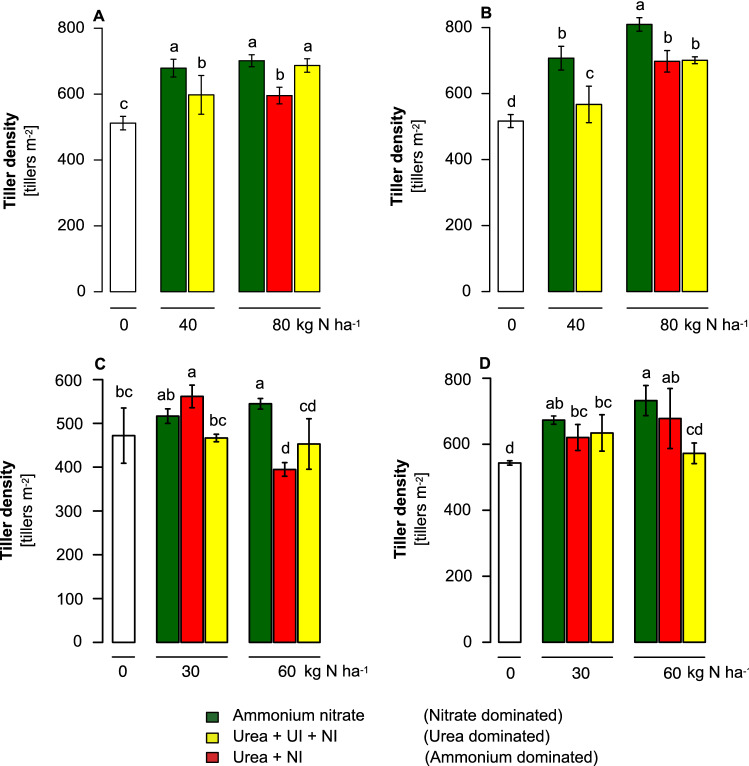
The influence of fertilizer N forms on tiller formation of field-grown winter wheat. Plants were grown either on the site Langenstein (**A**,**C**) or Dörrhof (**B**,**D**) in the growth season 2005/2006 (**A**,**B**) or 2006/2007 (**C**,**D**). Tiller density was determined at the end of vegetative development (BBCH 31) of winter wheat supplemented with 40 or 80 kg N ha^−1^ as starter dressing in 2006 and 30 or 60 kg N ha^−1^ as starter dressing in 2007 in the form of urea plus urease inhibitor (UI) and nitrification inhibitor (NI), providing urea-dominated N supply, urea plus nitrification inhibitor (ammonium-dominated N supply), or ammonium nitrate (nitrate-dominated N supply). Bars represent means ± SD, n = 4 independent replicate plots. Different letters indicate significant differences among fertilizer treatments at p < 0.05 by Tukey’s test.

We then verified the applicability of employing different fertilizer N forms for the control of tiller number in field-grown winter wheat. Wheat possesses a lower tillering potential than barley but shares with barley many regulatory mechanisms in tiller development^29,31–33^. Wheat was grown in four environments, i.e., at two sites in Germany in two subsequent years with substantial differences in temperature regime and precipitation (Supplementary Fig. 3 and 4). For the starter dressing in early spring, fertilizer N was supplied either (i) as fully stabilized urea, i.e., co-supplied with urease inhibitor and nitrification inhibitor, (ii) as urea plus nitrification inhibitor to provide mainly ammonium-N resulting from the rapid degradation of urea, or (iii) as ammonium nitrate, which was considered as predominant nitrate fertilization due to rapid adsorption of ammonium to the soil matrix, as a consequence of the high cation exchange capacity and the nitrification of ammonium. A pure nitrate fertilizer was not supplied to avoid side effects arising from accompanying cations. Relative to unfertilized control plots, nitrate as the predominant N form in the starter dressing led to significantly higher tiller numbers at the supply of 40 kg N ha^−1^ on both sites and at 80 kg N ha^−1^ also at the Dörrhof site, which is of poorer soil quality than the Langenstein site (Fig. 4A,B). Although N fertilization with fully stabilized urea increased tiller number relative to unfertilized plots, it was less effective, yielding 14–140 tillers m^−2^ less than after fertilization with nitrate. At the high N dose, application of urea only with nitrification inhibitor resulted in a similar tiller number as urea with both inhibitors. At harvest, however, total grain yield was higher in plants supplied with 80 kg ha^−1^ fully stabilized urea than in plants fertilized with ammonium nitrate (Supplementary Fig. 5A,B); this was not related to higher kernel weights (Supplementary Fig. 6A,B). Instead, climate data indicated that after a cold winter, the tillering period became relatively dry with rapidly rising temperatures in March and April 2006 (Supplementary Fig. 3A). The high number of additionally formed tillers under ammonium nitrate could not be fully developed to spike-bearing tillers and were mostly aborted. The stronger N form effect at higher fertilizer dose indicated that relative to mineralized soil N, a larger proportion of fertilizer N was required to significantly influence tiller formation.

In the subsequent year 2006/2007, which was characterized by a mild winter, a drought period at the end of the tillering phase in April but moderate temperatures with high precipitation during flowering and the grain filling period (Supplementary Fig. 4), ammonium nitrate-fertilized wheat plants also generated higher tiller densities than those fertilized with fully stabilized urea (Fig. 4C,D). Application of fully stabilized urea failed to increase tiller number considerably beyond the level of unfertilized control plants, suggesting slower conversion and/or utilization of urea-N. On both sites, lower tiller numbers in the two urea treatments did not translate into higher grain yield (Supplementary Fig. 5C,D). In contrast, on the less fertile soil at Dörrhof, the lower tiller density formed with 60 kg N ha^−1^ as fully stabilized urea translated into significantly lower grain yield. Thus, this low tiller density confined grain yield in combination with a limited number of grains per spike despite the humid grain-filling period (Supplementary Fig. 4B), leading to higher test weight of the urea-fertilized grains (Supplementary Fig. 6D). Taken together, in seven out of eight experimental scenarios, the provision of fully stabilized urea in the starter dressing led to lower tiller densities than supply with ammonium nitrate, although the size and significance of the difference in tiller number varied between the two N fertilizer regimes.

**Figure 5:**
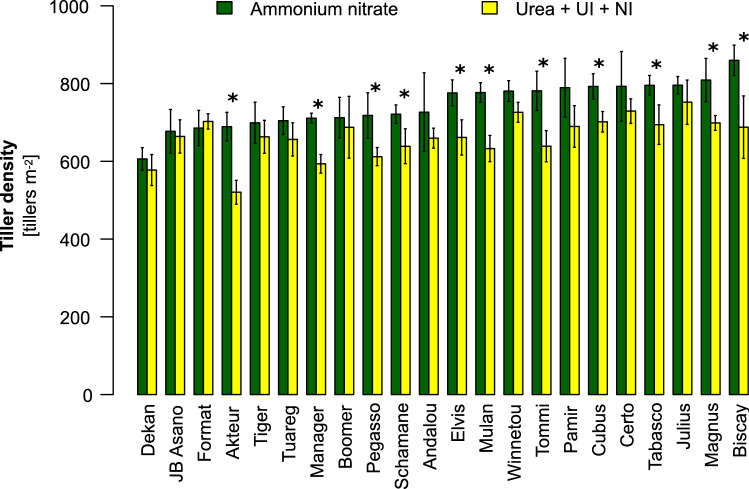
Genotypic variation of tiller formation in 22 field-grown winter wheat cultivars in dependence of the form of N nutrition. Tiller density was determined at the end of vegetative development (BBCH 31) of winter wheat plants supplemented with 80 kg N ha^−1^ as starter dressing in the form of urea plus urease inhibitor (UI) and nitrification inhibitor (NI), providing urea-dominated N supply, or ammonium nitrate (nitrate-dominated N supply). Plants were grown on the site Gatersleben in 2009/2010. Bars represent means ± SD, n = 4 independent replicate plots. Asterisks indicate significant differences among fertilizer treatments at p < 0.05 by Tukey’s test.

Based on the observation that wheat cultivars can differ in tiller formation^16,17^, we addressed whether the genetic variation of this phenotypic trait is also sensitive to the fertilized N form. We assessed a panel of 22 elite winter wheat cultivars in a separate trial and found in 11 cultivars a significantly higher tiller number under nitrate-dominated than under urea-dominated fertilization (Fig. 5). Ten of the remaining lines showed the same difference in trend. Under either N fertilizer regime, genotypic variation in tiller number mounted up to 200 tillers m^−2^, accounting for approx. 25% of maximum tiller number at BBCH 31. Thereby, tiller number of cultivars ranked differently in the two fertilizer regimes, indicating genotypic differences in the tillering response of a cultivar to the fertilized N form.

## Discussion

In cereal crops, the number of tillers per plant is a major yield component that is determined during late vegetative growth and is highly variable and dependent on the plant species and genotype as well as on growth conditions^17,18,29,34–36^. Tiller number is critical because weak tiller densities fail to exploit yield potential, while excess tillers get aborted or set grains that cannot be filled sufficiently^17,37^. Here, we show that nitrate as predominant N form enhances tiller number in hydroponics as well as in field trials, while the supply of the reduced nitrogen forms ammonium and urea decrease tiller formation despite efficient N delivery (Figs. 1, 2 and 4). Thereby, tiller formation is closely related to the root-to-shoot translocation of cytokinins, signalling the presence of the available N form to the shoot. The present study shows that tiller number in cereal crops can be controlled by the targeted choice of the fertilizer N form, which thus represents a simple and convenient management tool to manipulate this major yield component during vegetative crop development.

Three lines of evidence indicated that the translocation of cytokinins from roots to shoots is a major determinant for the control of tiller number but differentially affected by the supplied N form: (i) tiller number in barley plants supplied with different ratios of nitrate to urea correlated most closely with cytokinin translocation rates and were less associated with nutritional disorders (Fig. 1; Supplementary Fig. 1, 2); (ii) external supply of the synthetic cytokinin analog BA enhanced tiller number in barley, however, only when plants were grown with nitrate but not with ammonium or urea (Fig. 2); and (iii) translocation rates of the cytokinin analog BAR in the xylem sap increased in the presence of nitrate but not in the presence of ammonium or urea (Fig. 3). Possible reasons for partially contradictory or missing relations between N forms and cytokinin levels, as reported previously in wheat^19,20^, might be related to lacking consideration of N form-dependent pH effects in the soil or the partial conversion of non-stabilized N forms during the experiments. Here, nutrient solution pH was buffered, and urea was stabilized by PPD, which by itself did not provoke adverse growth effects in hydroponics (Fig. 1; Supplementary Fig. 1).

Enhanced de-novo synthesis of cytokinins in response to nitrate, as suggested from the identification of nitrate-inducible isopentenyl-transferase genes in Arabidopsis roots^25,38^, can only in part but not fully explain the N form-dependent differences in cytokinin translocation rates observed here. A key reaction in the synthesis of the physiologically most active form trans-zeatin and its transport form trans-zeatin riboside is the transhydroxylation of isopentenyladenine-type cytokinins by cytochrome P450 enzymes because only trans-zeatin but not isopentenyladenine can promote shoot growth and shoot branching^39^. This fully agrees with the poor N form-dependent response of isopentenyladenine and isopentenyladenosin in the xylem sap of nitrate- and urea-supplied plants (Fig. 1E). It will be thus important to find out in future whether expression of the cytochrome P450 orthologs catalyzing the transhydroxylation of isopentenyladenine-type cytokinins in barley responds to supplied N forms and is induced by nitrate.

Besides de-novo synthesis, we provide evidence that also the xylem loading step of cytokinins responds to the form of N supply. Only when roots were exposed to nitrate, externally supplied BA promoted tillering (Fig. 2). While this experiment proved the functionality of BA as a tillering-promoting signaling compound, this effect might also have been derived indirectly from enhanced biosynthesis of endogenous cytokinins in roots. This option was excluded by the external supply of BAR and its specific immune-detection in the xylem sap, which showed that exposure of roots to nitrate but not to ammonium or urea strongly enhances the accumulation of BAR in the xylem sap (Fig. 3). Thus, nitrate increased the xylem loading process of BAR, supposing that root uptake of BAR remained unaffected by the different N forms. In Arabidopsis, xylem loading of cytokinins is likely mediated by ABCG14, an ABC-type exporter localized in the plasma membrane of pericycle and other stelar root cells^40,41^. It will thus be interesting to elucidate whether the corresponding transporter in barley is subject to transcriptional regulation by N forms.

Based on these physiological experiments, we hypothesized that the differential effect of the N forms nitrate and urea on tiller formation in barley is also valid under field conditions. For the field trails, we used winter wheat, not only because of its higher agronomic importance but also to verify our hypothesis in another cereal crop species that has a lower tillering potential than barley^29^. Nonetheless, tiller formation in either species responds in a highly similar manner to environmental triggers, including photoperiod sensitivity or nutrient supply^16,29,31,32^. Also, major components in the genetic control of tillering are shared between wheat and barley^33^. In field-grown winter wheat, the supply of nitrate in the starter dressing enhanced tiller numbers mostly in a dose-dependent manner, whereas the stimulating effect of urea-derived N on tillering was significantly weaker at least in three out of four environments (Fig. 4A–D). The requirement for higher doses of fertilizer-nitrate to stimulate tillering was most likely due to the presence of reduced soil N forms, which are usually more abundant in fertile soils and after mild winters with higher mineralization of organic matter. Hence, the dose–effect of nitrate was apparently stronger on the less fertile site at Dörrhof with lower organic matter content (Supplementary Fig. 4B). Further promoted by higher precipitation during the grain-filling period, the elevated tiller number at Dörrhof in 2006/2007 also translated into higher grain yield (Fig. 4D, Supplementary Fig. 5D). In the other cases, tiller abortion may have exerted a significant impact, increasing in importance for a cultivar’s yield performance when the generative growth period falls dry^42^. Indeed, in the drier season 2005/2006 (Supplementary Fig. 3), urea-fertilized plants with lower tiller numbers tended to yield better (Fig. 4A,B; Supplementary Fig. 5A,B), which may be due to lower water consumption of a less dense plant stand. By contrast, the low tiller number of urea-fertilized plants at Dörrhof in 2006/2007 (Fig. 4D) most likely prevented achieving the full yield potential despite an increase in test weight (Supplementary Fig. 6D). The tillering-controlling effect of the fertilized N form was further confirmed in another trial that also addressed the question of the genotypic variation in this response. While the genotypic variation in tiller number was similarly high under fertilization of either N form, relative differences among cultivars differed substantially in the two fertilizer treatments (Fig. 5). This indicated a considerable impact of the genetic constitution of a wheat cultivar on the tillering response to either N form. To what extent genotypic control over N-responsive tiller formation can be exploited in crop breeding will depend on the firm implementation of defined N fertilization strategies and the identification of genomic loci associated with differential tillering responses to either N form in larger gene pools.

The present investigations give reason to consider more intensively the effects of fertilizer N forms on plant development, thus N fertilizer choice within current mineral N fertilizer practices. Based on this work, we recommend employing N fertilizer forms in the starter dressing that will adjust tiller number according to the tillering already achieved. If winter cereals have suffered from a short vegetation period due to late sowing or a strong winter and produced low tiller numbers, nitrate-based starter dressing will favor subsequent tillering. In contrast, if plants have passed a mild winter or an early sowing date that allowed more intensive tiller formation, the application of stabilized urea or ammonium fertilizers will suppress further tillering. Under dry or unfavorable conditions of generative growth, lower tiller numbers will favor grain setting and grain filling. Thus, we propose that cytokinin-mediated signaling effects caused by using different N forms can be employed as an additional management tool to control tiller number and thus a major yield component, even though the final impact on grain yield will further depend on tiller abortion and other cytokinin-dependent developmental processes like leaf senescence. Considering tiller-controlling N forms is particularly relevant for future fertilization strategies, which must respond with higher versatility to more extreme weather conditions in a changing global climate.

## Material and methods

### Plant material and growth conditions

Summer barley (cv. Henni) seeds were germinated in the dark for 4 days in CaSO_4_-saturated quartz sand before transfer to nutrient solution containing 1 mM KH_2_PO_4_, 1 mM MgSO_4_, 250 µM K_2_SO_4_, 250 µM CaCl_2_, 100 µM Na-Fe-EDTA, 50 µM KCl, 30 µM H_3_BO_3_, 5 µM MnSO_4_, 1 µM ZnSO_4_, 1 µM CuSO_4_, and 1 µM NaMoO_4_, pH was buffered at 6.6 by Ca(HCO_3_)_2_. Nitrogen was supplied at a concentration of 0.5 mM N as nitrate, ammonium, urea or mixtures of them. To suppress exogenous urea degradation by urease liberated from decaying root cells, all treatments were supplied with 75 µg L^−1^ of the urease inhibitor phenylphosphorodiamidate (PPD)^43,44^. The nutrient solution was renewed once a week during the first week and every 3 days for the following weeks. Plants were grown hydroponically under non-sterile conditions in a growth cabinet under the following conditions: 16/8 h light/dark; light intensity 280 µmol m^−2^ s^−1^; temperature 25 °C/20 °C and 60% humidity. Tillers were counted at the 7-leaf stage before plants were harvested, and shoots and roots freeze-dried and weighed separately. Only visible tillers were counted, but not the emerging tillers that were still behind the sheath.

### Cytokinin supplementation studies

In a long-term experiment, barley plants were grown hydroponically until the 3-leaf stage under 0.25 mM NH_4_NO_3_ as sole N source. Then, plants were transferred to 0.5 mM KNO_3_, 0.38 mM KNO_3_ + 0.06 mM urea (25% urea-N) or to 0.12 mM KNO_3_ + 0.19 mM urea (75% urea-N) and supplemented with 10 µM 6′-benzylaminopurine (BA). Dry weight and tiller number was determined at the 7-leaf stage.

In a short-term experiment, barley plants were grown on 0.25 mM NH_4_NO_3_ as sole N source until the 5-leaf stage before being exposed to N-free nutrient solution for 24 h. Subsequently, plants were transferred to either 0.5 mM N in the form of KNO_3_, urea or (NH_4_)_2_SO_4_, and after further growth for 36 h 10 µM 6′-benzylaminopurine riboside (BAR) was added to the nutrient solution. 12 h later, shoots were decapitated at the hypocotyl and xylem bleeding sap was collected as described above.

### Ammonium and urea determination

About 50 mg of freeze-dried and ground plant tissue were mixed with 1 ml 10 mM ice-cold formic acid in an Eppendorf tube, vortexed and centrifuged at 16,000 g (4 °C) for 15 min to recover the supernatant. Ammonium was determined according to Husted et al. (2000)^45^. 30 µL of the sample were injected into a column-less HPLC system running with a 3 mM o-phthalaldehyde- and 10 mM mercaptoethanole-containing reaction buffer (100 mM K_2_HPO_4_, pH 6.8). The reaction capillary was incubated at 80 °C and the samples were read at 410 nm (excitation) and 470 nm (emission).

Urea concentrations were determined based on a colorimetric reaction used by Kyllingsbaek (1975)^46^. Approx. 50 mg freeze-dried plant tissues were milled and suspended in 1 ml of cold 10 mM formic acid. After centrifugation at 13,200 rpm and 4 °C for 15 min, 30 μl of the supernatant were incubated with 1 ml of a colour development reagent (4.6 mM diacetylmonoxime, 1.28 mM thiosemicarbazide, 6.6% H_2_SO_4_, 14.6 µM ferric chloride hexahydrate and 0.006% ortho- phosphoric acid) at 99 °C for 15 min, and then cooled down at 4 °C for 5 min. Absorbance at 540 nm was measured with a photometer. The ureides allantoin, ornithine, arginine and uric acid did not interfere with urea determinations, however, other ureides were not tested.

### Collection of xylem bleeding sap

Stems of barley plants were cut approx. 10 mm above the hypocotyl before the cut surface was rinsed with bidistilled water and blotted dry with kimwipes. A silicon tube (diameter 1–4 mm, length 40 mm) was pulled over the cut stem surface, and xylem bleeding sap was collected during a period of 4 h. To increase the build-up of root pressure, the nutrient solution was supplemented with 0.5 mM K_2_SO_4_. Xylem bleeding sap of approx. 20 plants was pooled into one biological replicate, while three replicates per treatment were taken.

### Cytokinin and K determinations

Cytokinins in the xylem sap were determinated according to Bangerth (1994)^47^. The pH of the samples was adjusted to 8, and samples were purified by polyvenylpyrrolidone (PVPP) chromatography. The sample pH was then adjusted to pH 3 and samples were transferred to a methanol-activated C-18 Sep Pak cartridge (Walters, Milford, Mass. USA), which is able to bind cytokinins. The cartridge was rinsed with 15% (v/v) methanol in 0.1 M acetic acid and cytokinins were eluted by increasing methanol concentrations in the eluent. First zeatin and zeatin-riboside were eluted with 4 ml of 30% (v/v) methanol in 0.1 M acetic acid and then isopentenyl-adenine (i-Ado) and isopentenyl-adenosine (i-Ade) were eluted with 80% (v/v) methanol in 0.1 M acetic acid. The samples were vacuum evaporated and the cytokinins were determined by radioimmunoassay (RIA)^48^. All immunoassays were performed in triplicates. K concentrations were determined in the raw xylem exudate samples by flame photometry.

### Field trials

Field trials were conducted on a loamy silt under 488 mm annual rainfall in Northeastern Germany (site Langenstein) with the winter wheat cultivar Cubus or on a loamy clay under 720 mm annual rainfall (site Dörrhof) in Southern Germany with the winter wheat cultivar Schamane, the latter being better adapted to climate condition at the site Dörrhof. Wheat was sown and fertilized with 30 kg ha^−1^ N as ammonium sulfate in October. In March, plots were fertilized with urea coated with the urease inhibitor *N*-(*n*-butyl)thiophosphoric tiamide (NBPT), AGROTAIN, via spray application to granules, urea plus nitrification inhibitor (dicyandiamide and triazole) as commercial product or ammonium nitrate at a rate of 40 or 80 kg ha^−1^ N in 2006 and 30 or 60 kg ha^−1^ N in 2007. This way of N fertilization circumvented the application of accompanying salts and thus the application of additional fertilizers to balance possible effects of accompanying salts. All fertilizers were applied in granular form. At BBCH 31/32, 60 kg N ha^−1^ and at BBCH 39, 40 kg N ha^−1^ of ammonium nitrate was applied. Other treatments followed local agricultural practice required for a yield level of 10 t ha^−1^ at the site Langenstein and 9 t ha^−1^ at the site Dörrhof. The screening of 22 commercial varieties of winter wheat was conducted on a loamy silt under 592 mm annual rainfall in Northeastern Germany (site Gatersleben) in the growth period 2009/2010. In March, plots were fertilized with urea coated with NBPT or ammonium nitrate at a rate of 80 kg N ha^−1^. In all experiments, tiller number was determined at BBCH 31, while other yield components were determined before or at harvest. Crops were sown in a fully-randomized block design with four replicates per treatment.

## Supplementary information

Supplementary Figures.

## Abbreviations

PPD: Phenylphosphoro-diamidate
BA: 6-Benzylaminopurine
BAR: 6-Benzylaminopurine riboside
