# Supplementary material for: Modulating tiller formation in cereal crops by the signalling function of fertilizer nitrogen forms
Source: Sci Rep. 2020 Nov 25;10:20504. doi: 10.1038/s41598-020-77467-3 (PMC7689482; doi:10.1038/s41598-020-77467-3)
Supplement: Supplementary file 1 — Supplementary Figures. [file 41598_2020_77467_MOESM1_ESM.pdf]

Supplemental Information

Modulating tiller formation in cereal crops by the signalling function of fertilizer nitrogen forms

Bernhard Bauer[1,2], Nicolaus von Wirén[1*]

[1]Molecular Plant Nutrition, Leibniz-Institute of Plant Genetics and Crop Plant Research (IPK), Corrensstr. 3. 06466 Gatersleben, Germany

[2]Crop Production and Crop Protection, Institute of Biomass Research, University of Applied Science Weihenstephan-Triesdorf, Markgrafenstrasse 16, 91746 Weidenbach, Germany

*Corresponding author:
Nicolaus von Wirén
e-mail: vonwiren@ipk-gatersleben.de

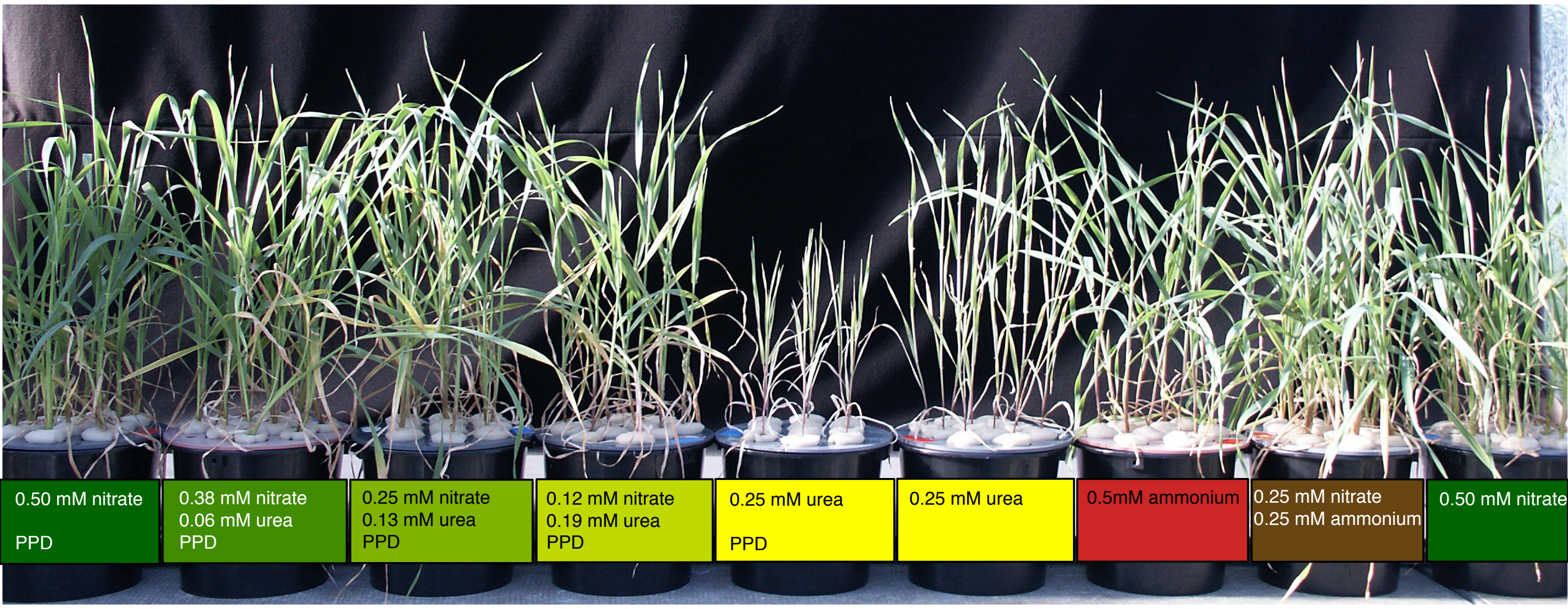

Supplementary Figure 1. Growth phenotype of barley shoots as affected by the supply of different nitrogen forms. Five d-old barley seedlings were transferred to nutrient solution containing the following N sources: 0.5 mM KNO_3, 0.38 mM KNO_3 + 0.06 mM urea (75:25% N), 0.25 mM KNO_3 + 0.13 mM urea (50:50% N), 0.12 mM KNO_3 + 0.19 mM urea (25:75% N), 0.25 mM urea, 0.25 mM NH_4NO_3. The pH was buffered at pH 6.6 by $Ca(HCO_3)_2$, and wherever indicated 75 µg L^{-1} of the urease inhibitor PPD was added. The photograph was taken after 40 days.

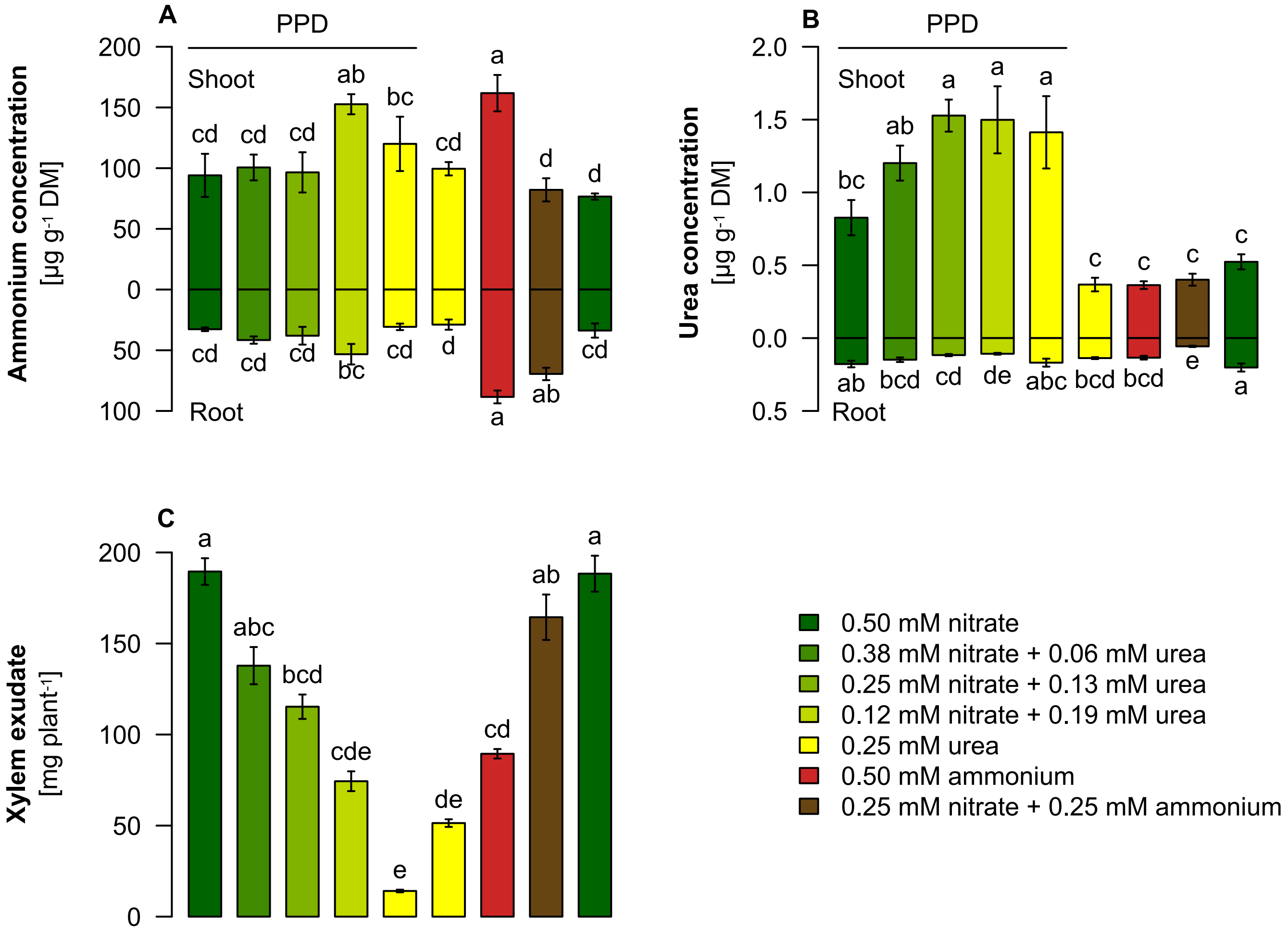

Supplementary Figure 2. Influence of the supply of different nitrogen forms on ammonium and urea concentrations in shoots and roots and on the xylem exudation rate. **(A)** Ammonium concentration and **(B)** urea concentration in shoots and roots. **(C)** Volume of collected xylem bleeding sap during 4 h. Barley seeds were germinated on $CaSO_4$-saturated quartz sand and after 5 d transferred to nutrient solution containing 0.5 mM KNO_3, 0.38 mM KNO_3 + 0.06 mM urea (75:25% N), or 0.25 mM KNO_3 + 0.13 mM urea (50:50% N), 0.12 mM KNO_3 + 0.19 mM urea (25:75% N), or 0.25 mM urea. The pH was buffered at pH 6.6 by $Ca(HCO_3)_2$. PPD indicates supplementation of 75 µg L^{-1} of the urease inhibitor phenylphosphoro-diamidate. Bars represent means ± SD, n = 3. Different letters indicate significant differences among means at $p < 0.05$ by Tukey's test.

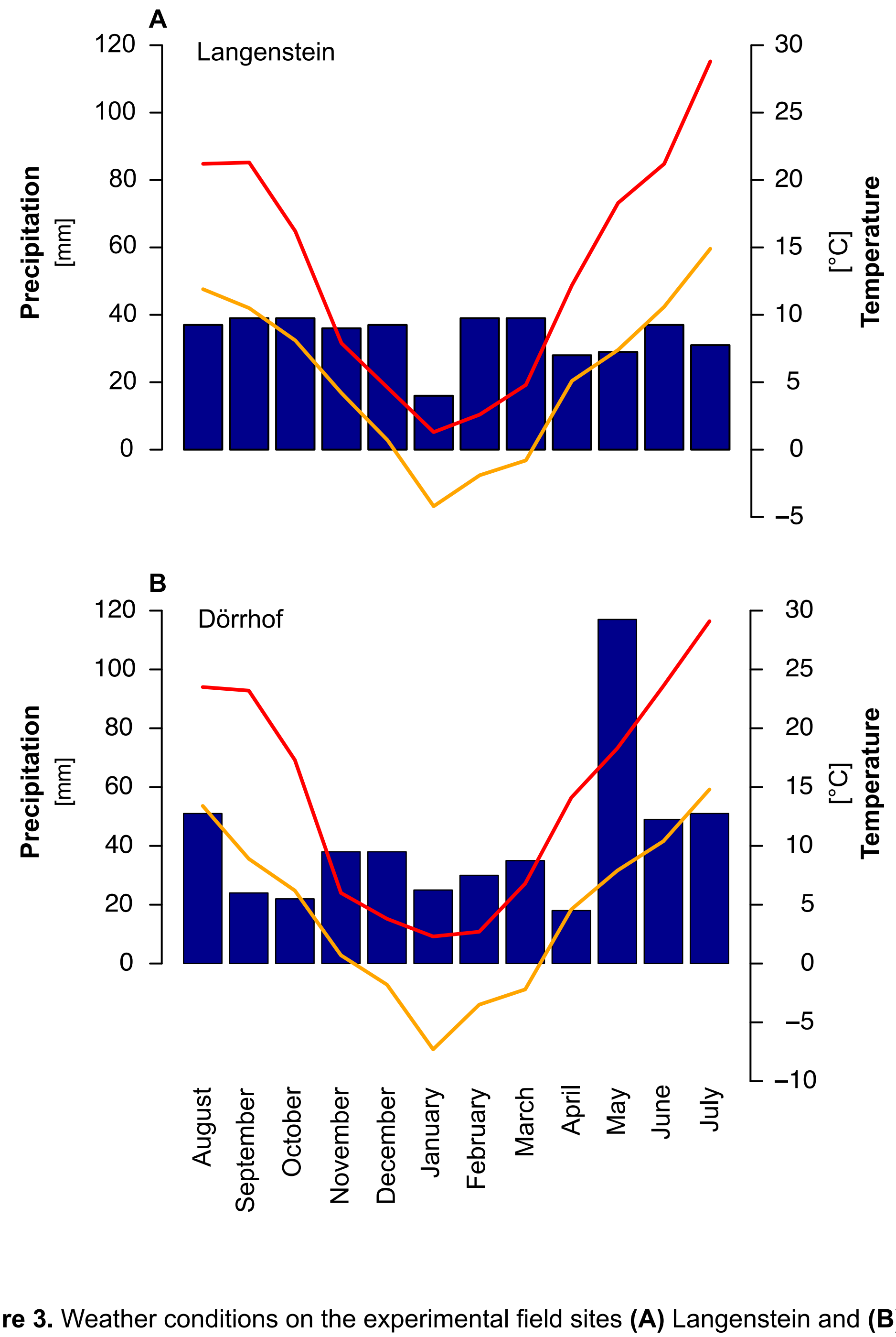

Supplementary Figure 3. Weather conditions on the experimental field sites **(A)** Langenstein and **(B)** Dörrhof as recorded by the local weather station. Bars represent precipitation and curves represent maximum (red) and minimum (orange) average temperature of each month in the experimental season 2005/2006.

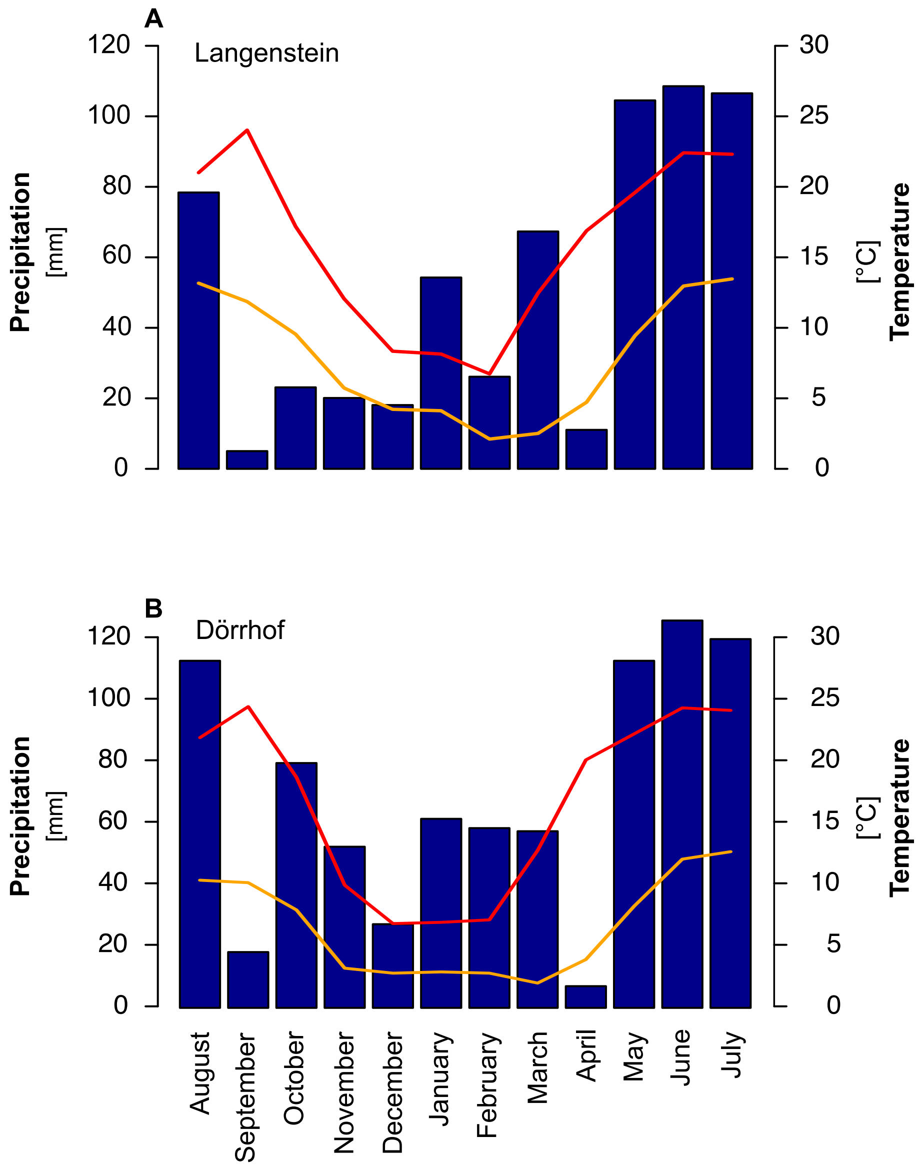

Supplementary Figure 4. Weather conditions on the experimental field sites **(A)** Langenstein and **(B)** Dörrhof as recorded by the local weather station. Bars represent precipitation and curves represent maximum (red) and minimum (orange) average temperature of each month in the experimental season 2006/2007.

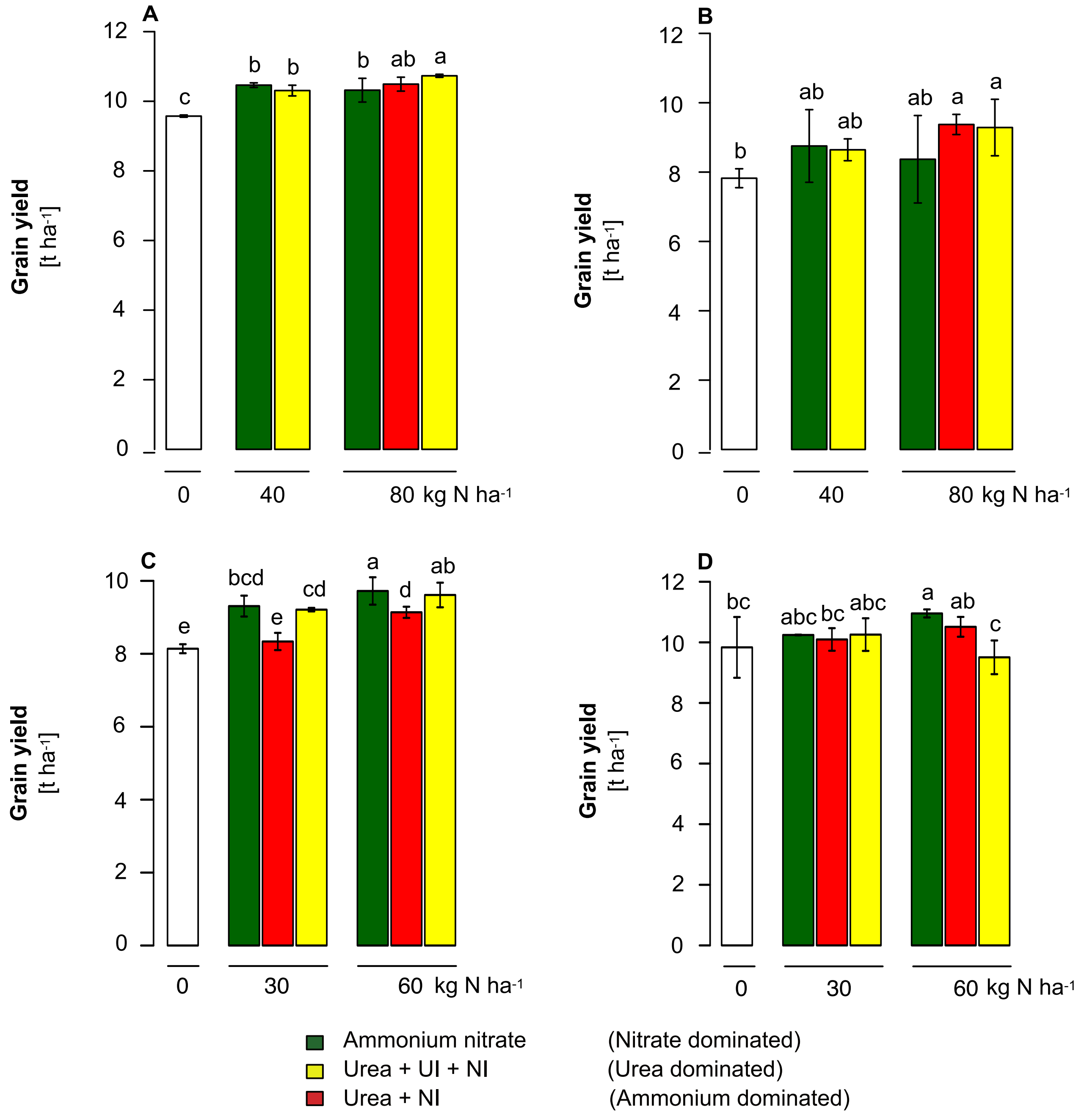

Supplemental Figure 5. The influence of fertilizer N forms on grain yield of field-grown winter wheat. Plants were grown either on the site Langenstein **(A, C)** or Dörrhof **(B, D)** in the growth seasons 2005/2006 **(A, B)** and 2006/2007 **(C, D)**. Grain yield was determined in mature wheat plants supplemented with 40 or 80 kg N ha^{-1} as starter dressing in 2006 and 30 or 60 kg N ha^{-1} as starter dressing in 2007 in the form of urea plus urease inhibitor (UI) and nitrification inhibitor (NI), providing urea-dominated N supply, urea plus nitrification inhibitor (ammonium-dominated N supply), or ammonium nitrate (nitrate-dominated N supply). Bars represent means ± SD, n = 4 independent replicate plots. Different letters indicate significant differences among fertilizer treatments at $p < 0.05$ by Tukey's test.

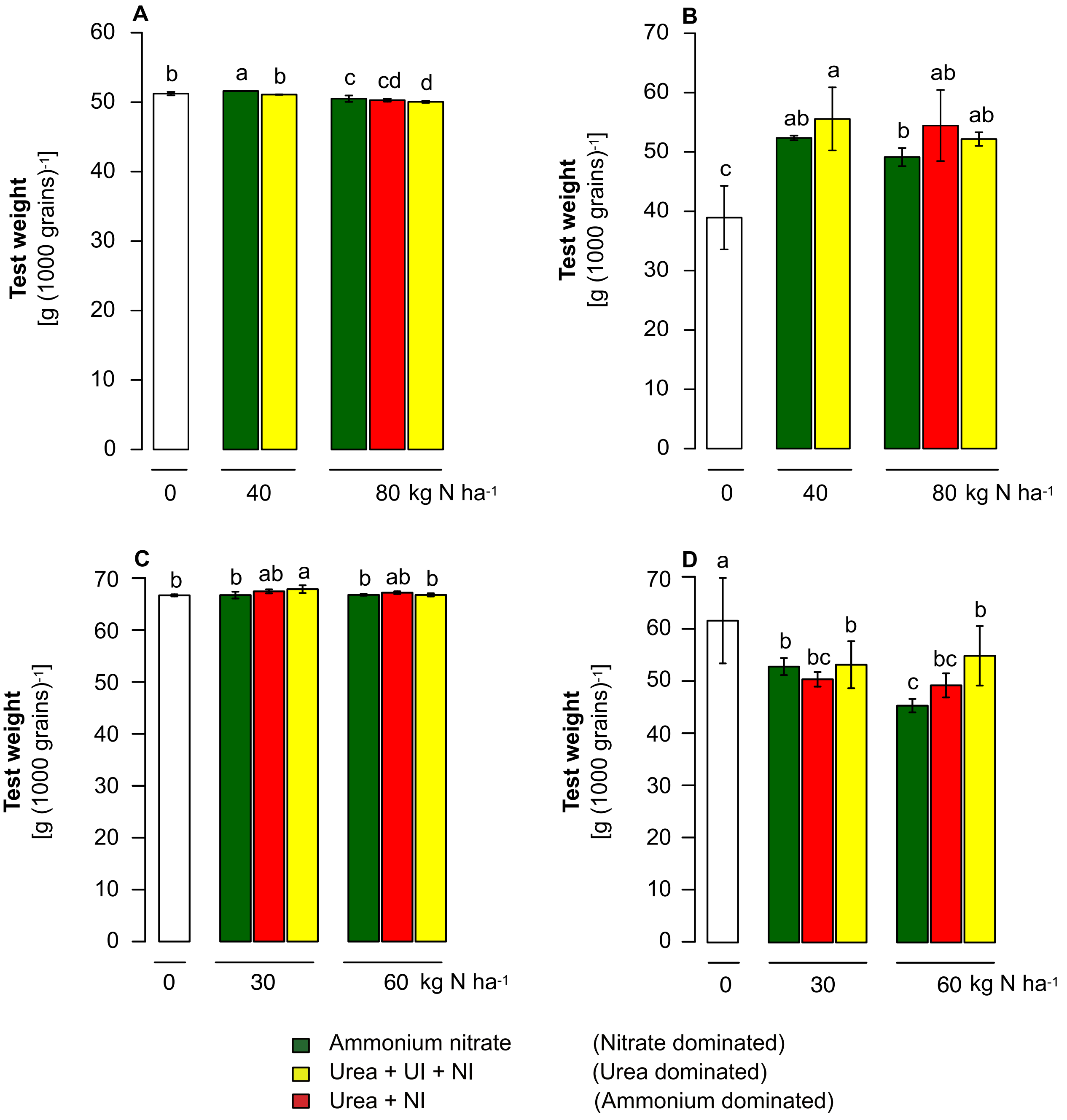

Supplemental Figure 6. The influence of fertilizer N forms on test weight of field-grown winter wheat. Plants were grown either on the site Langenstein **(A, C)** or Dörrhof **(B, D)** in the growth seasons 2005/2006 **(A, B)** and 2006/2007 **(C, D)**. Test weight (1000 kernel weight) was determined in mature and dried wheat plant supplemented with 40 or 80 kg N ha^{-1} as starter dressing in 2006 and 30 or 60 kg N ha^{-1} as starter dressing in 2007 in the form of urea plus urease inhibitor (UI) and nitrification inhibitor (NI), providing urea-dominated N supply, urea plus nitrification inhibitor (ammonium-dominated N supply), or ammonium nitrate (nitrate-dominated N supply). Bars represent means ± SD, n = 4 independent replicate plots. Different letters indicate significant differences among fertilizer treatments at $p < 0.05$ by Tukey's test.
